# *Mycobacterium avium* complex: Adherence as a way of life

**DOI:** 10.3934/microbiol.2018.3.428

**Published:** 2018-06-12

**Authors:** Joseph O. Falkinham

**Affiliations:** Department of Biological Sciences, Virginia Tech, Blacksburg, Virginia 24061, USA

**Keywords:** mycobacteria, *M. avium* complex, MAC, adherence, hydrophobic, biofilm, disinfectant, antibiotic, methylobacteria

## Abstract

*Mycobacterium avium* complex (MAC) organisms are waterborne, opportunistic pathogens whose source is natural waters and soils and proliferates and persists in premise plumbing, for example household and hospital plumbing. *M. avium* complex and other environmental mycobacteria grow slowly, not because their metabolism is slow, but because they synthesize long chain (C_60_–C_80_) fatty acids that make up its hydrophobic and impermeable outer membrane. There are costs and benefits to the presence of that lipid-rich outer membrane. One benefit is that cell-surface hydrophobicity drives *M. avium* complex cells to adhere to surfaces to reduce their interaction with charged ions in suspension; they are likely “biofilm pioneers”, adhering to a wide variety of surface materials. The result is that the slow-growing *M. avium* complex cells (1 gen/day at 37 °C) will not be washed out of any flowing system, whether a stream or plumbing in the built environment. Although the slow permeation of nutrients in *M. avium* complex organisms limits growth, they are also resistant to disinfectants, thus increasing their survival in water distribution systems, premise plumbing, and medical equipment. There are three components to the antimicrobial resistance of *M. avium* complex in biofilms: (1) innate resistance due to the hydrophobic, impermeable outer membrane, (2) residence in a matrix of extracellular polysaccharide, lipid, DNA, and protein that prevents access of antimicrobials to *M. avium* cells, and (3) an adaptive and transient increased resistance of biofilm-grown *M. avium* cells grown in biofilms. As expected *M. avium* in biofilms will display neutral, antagonistic, or beneficial interactions with other biofilm inhabitants. *Methylobacterium* spp., the common pink-pigmented, waterborne bacteria compete with *M. avium* for surface binding, suggested an approach to reducing *M. avium* biofilm formation and hence persistence in premise plumbing.

## 1. Introduction

*Mycobacterium avium* complex organisms and other nontuberculous mycobacteria (NTM) are waterborne opportunistic pathogens with a strong propensity for surface adherence and biofilm formation. *M. avium* complex and other NTM are slow growing, hydrophobic, and impermeable to nutrients due to the presence of a lipid-rich outer membrane. Although those features result in slow mycobacterial growth, their metabolism is rapid and robust and the hydrophobic impermeable outer membrane confers antibiotic- and disinfectant-resistance. *M. avium* complex and NTM are found in natural waters and also colonize, grow, and persist in drinking water distribution systems and premise plumbing. Their presence in plumbing in hospitals is associated with life-threatening nosocomial infections. Efforts to eradicate *M. avium* complex and NTM are thwarted by their preference for surface adherence and biofilm formation. This review identifies those *M. avium* complex features in hopes that methods to reduce numbers and hence exposure and infection of those opportunistic pathogens can be developed.

## 2. *Mycobacterium avium* characteristics

*M. avium* complex, including *M. avium, M. intracellulare*, and *M. chimaera* are opportunistic premise plumbing pathogens that share many environments with humans. In short, humans are surrounded by mycobacteria [1]. Of present concern is that the incidence and prevalence of pulmonary disease caused by mycobacteria, predominantly *M. avium* complex in the United States, is increasing a rate of 5–10% annually [2]. Risk factors for *M. avium* complex pulmonary disease include: chronic obstructive pulmonary disease (COPD), bronchiectasis, a history of smoking, silicosis, and other occupation-related lung conditions [3]. Currently, women who are older (>60 years), taller (>5′8″), and slender (BMI < 22), first identified in 1989 [4], are at increased risk for *M. avium* complex pulmonary disease and make up the majority of infected individuals. Taller, slender, older men are also at increased risk for *M. avium* complex pulmonary disease. The treatment of *M. avium* complex pulmonary disease requires a long-term course of multiple antibiotics [5]. However, if patients can tolerate the side effects of the multiple antibiotics, they can be free of disease symptoms. Unfortunately, recurrence of symptoms and reappearance of mycobacteria in sputum is high; namely, 25–50% of patients depending on the study [6],[7]. Recurrence of symptoms coincides with either recovery of the same *M. avium* complex strain or another *Mycobacterium* spp. from sputum samples. Young children (18 months to 5 years) are susceptible to *M. avium*-caused cervical lymphadenitis [8]. As these youngsters have erupting teeth with the associated trauma to the gums, cervical lymphadenitis is consistent with environmental acquisition of *M. avium* and infection of the local cervical lymph nodes. The best treatment for *M. avium* cervical lymphadenitis is surgical excision of the infected lymph node(s) [8].

Infection sources of *M. avium* complex includes soils, especially peat-rich, pine forest soils [9], natural waters (e.g., streams, rivers, lakes) [10], and drinking water in distribution systems [11] and premise plumbing [12]. Demonstration that the DNA fingerprints of *M. avium* isolates from a showerhead matched those of an *M. avium*-infected patient who used the shower [13] was followed by a DNA-based survey of bacteria in United States showerheads. That survey showed that 70% of U.S. showerheads, had *Mycobacterium* spp. and of those 30% had *M. avium* [14]. As it would be understood that the majority of the showerheads came from homes of individuals that were not infected by *M. avium*, the results demonstrated that humans are surrounded by *Mycobacterium* spp., including *M. avium*. Hospital plumbing and water-containing medical equipment, such as bronchoscopes [15], can become colonized by *M. avium* complex organisms and thereby transferred to patients during examination or sample withdrawal. The most dramatic example of mycobacterial colonization, hospital transmission, and infection was disclosed in October 2015. *M. chimaera*, was shown to have colonized heater-coolers using during cardiac surgery and patients were infected as a result of the aerosolization of the *M. chimaera* cells during surgery [16].

The colonization and persistence of *M. avium* in drinking water in homes and hospitals is due to a number of factors, including: biofilm-formation [17], growth in drinking water [11],[18], resistance to disinfectants (e.g., chlorine) used in water treatment [19], growth at low concentrations of organic matter in drinking water [20], resistance to high temperatures (e.g., 50–65 °C), and growth at low oxygen concentrations (6–12% oxygen) [21].

The major determinant of *M. avium* complex ecology is the lipid and wax-rich outer membrane that surrounds the cells [22]. It is composed of long chain fatty acids (C_60_–C_80_) and makes up 30% of the entire cell mass. This outer membrane is responsible for *M. avium*'s slow growth (1 gen/day), as a substantial fraction of energy is diverted to synthesis of the lipids. In addition to high cell surface hydrophobicity—for example, a droplet of water will form a bead on the surface of *M. avium* cells collected by filtration—the outer membrane of *M. avium* is quite impermeable to nutrients [23]. The outer membrane presents us with a thought-provoking example of cost-benefit analysis. First, that impermeable membrane reduces the transport of nutrients into the cytoplasm [23] thus, in part, limiting the rate of growth; a clear cost. However, that impermeable membrane also makes *M. avium* cells quite resistant to disinfectants, like chlorine [19], and other anti-microbial compounds (e.g., metals) [24]; a clear benefit. That benefit means *M. avium* cells survive disinfection of drinking water and, in the absence of other microbial cells (killed by disinfectant), they can consume the remaining organic matter.

## 3. Biofilms as a selective environment

Biofilms are selective environments, placing demands on planktonic microbial cells. Adherence can be inhibited by surface toxicity, as proposed responsible for the few cells of *Pseudomonas aeruginosa* adhering to copper pipes [25]. A pre-existing microbial biofilm may stimulate, inhibit, or have no effect on adherence of a particular microbial species or type. For example, early studies of adherence relied upon “conditioning” a surface with protein (e.g., bovine serum albumin); without a conditioned surface a particular microorganism would not adhere and form a biofilm. In contrast, *M. avium* is a “biofilm pioneer”. Its hydrophobic surface drives its adherence to surfaces of all composition [17]. Further, as described below, *M. avium* compete for adherence to surfaces with another “biofilm pioneer”, the *Methylobacterium*, a genus of common waterborne bacteria that form a “pink slime” in showers, shower curtains, and bathtubs.

Persistence of a species in a biofilm places selective constraints on microbial cells. Due to the layers of cells embedded in an extracellular matrix, an *M. avium* complex biofilm comprised of lipid, protein, and DNA [26],[27], penetration of oxygen and nutrients is limited [28]. Thus, a species commonly found in biofilms must be able to grow under low oxygen and low nutrient conditions. There may be a unique challenge to non-mycobacterial cells in biofilms as it has been shown that mycobacterial biofilms contain free fatty acids [26],[27] and free fatty acids are antimicrobial. Another antagonistic interaction was shown by the observation that *Mycobacterium abscessus* was capable of degrading quinolone-based quorum-sensing molecules produced by *Pseudomonas aeruginosa* [29], thus inhibiting biofilm formation by the pseudomonad. Further, it has been shown that biofilms of mycobacteria contain a substantial proportion (∼10%) of mycobacterial cells that survive exposure to antibiotics at concentrations higher than the minimal inhibitory concentration [27]. Although the metabolic state of those persisting cells is unknown, the consequence is that mycobacterial cells in biofilms can survive antibiotic challenge. It is to be expected that further studies of biofilm microbiomes may lead to discover of co-metabolizing microbial partners that coexist in biofilms by virtue of cross-feeding nutrients. Further, such studies may lead to identification of “trophic trees” describing chemical interactions between microbial species in biofilms.

## 4. Biofilms and *M. avium*

Surface adherence and biofilm formation is a necessity for members of the *M. avium* complex; without adherence, the slow-growing *M. avium* complex cells would be washed away in any flowing system, like rivers and water pipes. *M. avium* complex residence in flowing systems is due to their high cell surface hydrophobicity. In fact, *Mycobacterium* spp. may be the most hydrophobic of bacterial cells [30]. It is likely hydrophobicity that drives *M. avium* cells to adhere to surfaces as that action reduces the surface area in contact with the positive and negative ions in water. As suggested above, *M. avium* cells may be “biofilm pioneers”, adhering to surfaces through hydrophobic interactions in advance of other microorganisms, thereby “conditioning” the surfaces for further microbial colonization. Hydrophobicity is such a strong determinant of the special distribution of *M. avium* complex and other environmental mycobacteria, that the number and density of surface-adherent cells far outnumbers the number of cells in suspension.

Three experimental observations document the preference of *M. avium* complex for surface adherence. First, numbers of *M. avium* complex cells in biofilms far surpass numbers in water. For example, common values for *M. avium* complex numbers in drinking water in household plumbing ranges from 100–1,000 colony-forming units (CFU)/mL [12]. In contrast, numbers on pipe surfaces in the same households is between 10,000–20,000 CFU/cm^2^ [12]. Second, reduction of turbidity of water entering drinking water treatment plants is an effective method to reduce *Mycobacterium* spp. and *M. avium* complex densities in drinking water [11]. This is due to the fact that mycobacteria from environmental waters enter water treatment plants attached to soil particles and a reduction in turbidity reduces not only particulates, but also the particulate-adherent *Mycobacterium* spp. and *M. avium* complex cells [11]. The third example comes from our development of disinfection protocols for reducing numbers of *M. chimaera* in heater-coolers. Each of the 2–10 liter water reservoirs of heater-coolers were inoculated with approximately 100 billion cells (10^11^ CFU) to produce a worst case scenario for any disinfection protocol. Surprisingly, rather than recovering the 15 million cells/mL as we expected, less than 1 million (>6%) were recovered (Falkinham, in preparation). Those missing cells had not been killed, but simply lost from suspension because of their adherence to the pipes, tubes, pump, and reservoir surfaces. The critical fact was that the adherent *M. chimaera* cells were in biofilms, and almost completely resistant to disinfectant. Although exposure to chlorine killed greater than 3-logs of suspended *M. chimaera* cells in the reservoir water of the heater-cooler, the majority of cells survived in the biofilms and rapidly (1 week) re-inoculated the reservoir water (Falkinham, in preparation). What is critically needed is a method to “disrupt” the biofilm to render cells more accessible to disinfectant. Such an approach involving enzyme-detergent formulations has been shown to increase removal of biofilm cells from biofilms [31],[32].

Realization that *M. avium* and other *Mycobacterium* spp. cells prefer surface adherence to residence in suspension have led us to change our sampling strategy for their recovery and enumeration from environmental samples. Although we can isolate, enumerate, identify, and type *M. avium* cells from drinking water samples in households and hospitals, we recover many more by sampling the biofilms on the pipes. This provides us with a higher sensitivity of detection of *Mycobacterium* spp. and also a better picture of the diversity of *M. avium* types and clonal variants. That, in turn, increases our ability to find exact fingerprint matches between patient and environmental isolates [13].

If the lipid- and wax-rich outer membrane was not enough to provide resistance to disinfectants, antibiotics, and other antimicrobials (e.g., heavy metals) [24], biofilm formation increases both disinfectant [33] and antibiotic-resistance [34]. This is consistent with observations of other biofilm-forming opportunistic premise plumbing pathogens such as *Pseudomonas aeruginosa*. The extracellular matrix and the layers of cells reduce penetration and diffusion of disinfectant or antibiotic to the mycobacterial cells [28]. However, that is not all. Cells of *M. avium* complex grown in biofilms yet recovered and mechanically-treated to yield single cell suspensions are significantly more disinfectant-resistant [33] or antibiotic-resistant [34] than cells grown and exposed in suspension. That increased resistance is adaptive and transient, for overnight growth of the biofilm-grown and resuspended cells yields cells with susceptibilities to either disinfectants or antibiotics that are the same as cells grown in suspension [33],[34]. The rapid loss of that adaptation to antimicrobial resistance of *M. avium* cells is not surprising as their metabolic rate is as high as that of *E. coli.* Slow mycobacterial growth (i.e., 1 gen/day at 37 °C) is not evidence of slow metabolism but rather due to the expenditure of a large fraction of energy on the synthesis of the C_60_–C_80_ long chain lipids and waxes. That rapid loss of adaptive antimicrobial resistance may prove to be a value in eradicating *M. avium* complex and other nontuberculous mycobacteria from premise plumbing, including hospital plumbing. The strategy would be to exposure mycobacterial biofilms to a disinfectant for a short period of time, allow for a period of time to lose the adaptive biofilm-induced resistance, and then re-expose. That approach might prove to be superior to eradication by long-term continuous exposure as continuous selective pressure would maintain the adaptive resistance.

## 5. Biofilm-surface interaction

*M. avium* complex cells show a hierarchy of short term adherence to surfaces [17]. In part, it is likely that some of the heterogeneity in results is due to employing a variety of strains whose identity is not thoroughly established due to the continuing discoveries of novel species; in particular, *M. chimaera* [35] and its separation from *M. intracellulare* [36]. However, before reviewing that data, it is important to understand the two separate and distinct steps of biofilm formation: adherence and growth of adherent cells to form a biofilm. Adherence is measured by exposing a surface to a suspension of cells for relatively short periods of time (1–6 h at 25 °C). For *M. avium* complex organisms, adherence is relatively rapid and does not require any “conditioning” of the surface and the increase in adherent *M. avium* complex cells reaches a plateau within 3–6 h at 25° C [17]. After 6 h adherence, the surfaces can be removed from the suspension, rinsed with sterile tap water, and the coupons incubated in sterile water or medium to measure the growth of cells (i.e., biofilm formation). If the surfaces are not removed from the cell suspension, increases in numbers of adherence cells are due to not only growth of adherent cells but also include increases in numbers due to continued adherence. A great number of investigations of mycobacterial biofilm formation have suffered from that defect leading to an inability to measure the rate of adherence and just report the rate of accumulation. Biofilm formation, for these slowly-growing bacteria (1 gen/day), appears to reach a maximum number of adherent CFU/cm^2^ by 21 days incubation at room temperature [17]. Specifically, by 21 days, surface densities ranged from 1.4 × 10^7^ (galvanized steel) to 5.5 × 10^3^ (copper) CFU/cm^2^ [17].

Cells of *M. avium* appear to have a hierarchy for adherence. The hierarchy for at least one strain of *M. avium* adherence to surfaces was: galvanized stainless steel, stainless steel, polyvinyl chloride plastic, glass, and copper [17]. Highly rough surfaces such as those of galvanized stainless steel accumulated up to 15,000 CFU/cm^2^ within 3 h at room temperature [17]. In the absence of independent measures of surface hydrophobicity and roughness, it is not known whether both are factors and to what extent. Surface topology (e.g., roughness) has been shown to influence *Mycobacterium abscessus* adherence to surfaces [37]. Other factors, such as metal composition, could be involved as well. High numbers of adherent *M. avium* cells on galvanized surfaces could be due to the fact that numbers of *M. avium* in natural waters are positively correlated with the concentration of zinc [38]. Likewise, as copper surfaces accumulated the lowest numbers of *M. avium* cells [17] and low numbers of adherent *M. avium* cells were found on copper pipes in a pilot distribution system harbored low numbers of *M. avium* and almost no other microbial cells [20]. Those observations are also consistent with the fact that *M. avium* cells are relatively resistant to copper compared to other bacteria [24]. Quite possibly, copper and zinc pipes are somewhat selective for *M. avium* adherence. On PVC surfaces, *M. avium* biofilm formation was influenced, but not totally dependent upon the presence of Ca^2+^, Mg^2+^, and Zn^2+^ ions in solution [39]. Two other *M. avium* characteristics have also been shown to promote adherence and biofilm formation; presence of surface glycopeptidolipids [40] and sliding motility [41].

## 6. Biofilm-microbial interactions

The salmon pink-pigmented methylobacteria are common premise plumbing inhabitants. *Methylobacterium* spp. are major colonists of shower curtains [42] and shower heads [14]. Like *M. avium*, methylobacteria readily form biofilms, likely due to their high cell surface hydrophobicity (Swetkowski and Falkinham, in preparation). In a study of showerhead biofilms, it was shown that methylobacteria were quite common in shower head samples collected across the United States [14]. More prevalent were the mycobacteria; but that was not too surprising. What was surprising was that when methylobacteria were present in shower head biofilms, mycobacteria were absent, and vice-versa [14]. A study of plumbing biofilms in homes of *M. avium* infected women in Wynnewood, Pennsylvania showed the same pattern; when methylobacteria were present in high numbers, *M. avium* was absent [43]. A laboratory study has further showed that the presence of an established (21 day) *Methylobacterium* spp. biofilm significantly inhibited the adherence of *M. avium* cells [44]. The fact that different portions of the plumbing of houses may either have *M. avium* and no *Methylobacterium* spp. or *Methylobacterium* spp. and no *M. avium*, have led us to the hypothesis that the two compete for adherence when the plumbing is first filled with water. We have no evidence that *Methylobacterium* spp. cells can dislodge *M. avium* cells in a biofilm.

As *M. avium* complex- or NTM-infected patients and their physicians are aware of relapse and reappearance of disease symptoms [6],[7], those patients want to avoid mycobacterial exposure in their homes. Fortunately, if they have pink slime in showers, showerheads, sinks, or taps, it is unlikely that they have *M. avium* or any other NTM and can avoid installing microbiological point-of-use filters. Thus, presence of pink-slime is a strong indicator of the absence of *M. avium* or any other NTM.

## 7. Questions and future directions

The perspective that follows is based on translating knowledge of mycobacterial ecology and physiology to improve patient outcomes. The following paragraphs briefly summarize possible mycobacterial research objectives focused on their preference to adhere, grow, and persist in biofilms in premise plumbing. They include: (1) identification of *Methylobacterium* spp. cell fractions able to inhibit adherence of *M. avium* cells, (2) developing protocols to “disrupt” biofilms, thereby releasing *M. avium* cells and increasing their exposure to disinfectants, (3) elucidating the molecular basis for *M. avium* adaptation to disinfectant-resistance as a result of growth in biofilms, and (4) identifying other waterborne microorganisms whose presence is linked to *M. avium* presence or absence.

Toward the objective of identification of *Methylobacterium* spp. cell fractions able to inhibit adherence of *M. avium* cells, we discovered that the viability of the *Methylobacterium* spp. cells in a biofilm was not required to inhibit *M. avium* adherence. In fact, killed whole cells in biofilms can inhibit *M. avium* adherence (Muńoz-Egea, personal communication). We have initiated a project to identify whether cell fractions of *Methylobacterium* spp. cells coating a pipe can inhibit *M. avium* adherence. *Methylobacterium* spp. cell fractions could be used as a probiotic for plumbing, to prevent *M. avium* colonization of premise plumbing and medical equipment. Again it is important to point out that we have no evidence that *Methylobacterium* spp. cells can dislodge *M. avium* from a biofilm. That means *Methylobacterium* spp. cells or cell fractions could not be employed to reduce *M. avium* in a pre-existing biofilm. However, an immediate application of this technology could be used to prevent *M. chimaera* colonization and biofilm-formation in heater-coolers by introducing killed *Methylobacterium* spp. cells or cell fractions when the heater-cooler is first filled with water and its operation tested. That would prevent *M. avium* or NTM colonization and biofilm-formation and thereby reduce the aerosolization of any *Mycobacterium* spp. cells.

It is generally acknowledged that microbial cells in biofilms are resistant to antibiotics, disinfectants, and other antimicrobial agents. Another project is to develop protocols to “disrupt” biofilms, thereby releasing *M. avium* cells and increasing their exposure to disinfectants. Such “disruption” has been disclosed in an article describing the use of enzyme-detergent formulations to remove biofilms [28]. Not only was the efficacy of such enzyme-detergent formulations shown to catalyze biofilm release from surfaces, but the data showed species-specific release [31],[32]. Specifically, a protease formulation released more *Bacillus* sp. biofilm compared to a polysaccharidase containing formulation. Conversely, the polysaccharidase-formulation was more effective at removing *Pseudomonas fluorescens* biofilms than was the protease formulation [31]. It would be of value to identify which type of enzyme-detergent formulations would be most effective in releasing *M. avium* biofilms.

The observations that *M. avium* cells grown in biofilms yet isolated as single-cell suspensions were transiently more resistant to chlorine [33] or antibiotics [34], has focused our attention on mechanisms of adaptation of *M. avium*. Unlike other bacteria that grow rapidly, *M. avium* grows slowly, not as a result of slow metabolism and energy generation, but because it expends a great deal of energy in synthesis of the thick, lipid-rich outer membrane. *M. avium* generates energy at the same rate as does *Escherichia coli*. It grows slowly because energy is diverted to C_60_–C_80_ lipid synthesis. Thus, upon exposure to an environmental stress such as disinfectant, antibiotic or elevated temperature, *M. avium* cells can induce protective proteins leading to survival. In rapidly growing bacteria, division occurs before protection and death ensues. Preliminary experiments in the lab have shown that *M. avium* grown at 42 °C have 10-fold higher levels of trehalose compared to those grown at 25 °C. As high trehalose concentrations have been associated with resistance to temperature, desiccation, toxic oxygen compounds, and salinity [45], induction of elevated levels of trehalose in *M. avium* could be induced by biofilm growth as well as temperature.

Finally, evidence that *Methylobacterium* spp. cells could inhibit *Mycobacterium* spp. adherence to surfaces, came from studies of the microbiomes of showerheads [14]. As the samples analyzed came from showerheads across the United States [14], the observation was global and not just due to a particular set of local water conditions. That fact stimulated our interest and further exploration resulting in the data presented above. Further mining of the data in Feazel et al. [14] or additional analyses of the microbiomes of hospital plumbing, might lead to discovery of other *M. avium* antagonists or heretofore undiscovered microbial species that aid establishment of *M. avium* in premise plumbing.
